# Discovery and visualization of miRNA–mRNA functional modules within integrated data using bicluster analysis

**DOI:** 10.1093/nar/gkt1318

**Published:** 2013-12-18

**Authors:** Kenneth Bryan, Marta Terrile, Isabella M. Bray, Raquel Domingo-Fernandéz, Karen M. Watters, Jan Koster, Rogier Versteeg, Raymond L. Stallings

**Affiliations:** ^1^Department of Molecular and Cellular Therapeutics, Royal College of Surgeons in Ireland, York House, York Street, Dublin 2, Ireland, ^2^National Children’s Research Centre, Our Lady’s Children’s Hospital, Crumlin, Dublin 12, Ireland and ^3^Department of Oncogenomics, Academic Medical Center, University of Amsterdam, 1100 DE Amsterdam, The Netherlands

## Abstract

MicroRNAs (miRNAs) are small non-coding RNA molecules that regulate gene expression at a post-transcriptional level. An miRNA may target many messenger RNA (mRNA) transcripts, and each transcript may be targeted by multiple miRNAs. Our understanding of miRNA regulation is evolving to consider modules of miRNAs that regulate groups of functionally related mRNAs. Here we expand the model of miRNA functional modules and use it to guide the integration of miRNA and mRNA expression and target prediction data. We present evidence of cooperativity between miRNA classes within this integrated miRNA–mRNA association matrix. We then apply bicluster analysis to uncover miRNA functional modules within this integrated data set and develop a novel application to visualize and query these results. We show that this wholly unsupervised approach can discover a network of miRNA–mRNA modules that are enriched for both biological processes and miRNA classes. We apply this method to investigate the interplay of miRNAs and mRNAs in integrated data sets derived from neuroblastoma and human immune cells. This study is the first to apply the technique of biclustering to model functional modules within an integrated miRNA–mRNA association matrix. Results provide evidence of an extensive modular miRNA functional network and enable characterization of miRNA function and dysregulation in disease.

## INTRODUCTION

MicroRNAs (miRNAs) are small non-coding RNA molecules, ∼18–25 nt in length when mature, that regulate messenger RNA (mRNA) expression at a post-transcriptional level. This occurs chiefly through the binding of the seed region (nucleotides 2–8) of an miRNA, as part of the RNA-induced silencing complex, to complementary sequences in the 3′ UTR of a target mRNA followed by subsequent degradation and/or translational inhibition of the mRNA transcript. miRNA post-transcriptional regulation was first described in the context of an unusual non-coding RNA, *lin-4*, that regulated larval development in *C. elegans* (1). Gradually it became apparent that this mode of regulation was widespread, occurring across diverse cellular functions and species (2–5). miRNAs have now been implicated in a wide range of biological processes including development, cell growth and cell division (6). Sixty percent of human coding genes possess conserved target sites for miRNAs (7,8). Given the systemic importance of miRNAs, it is unsurprising that their dysregulation has been shown to be a factor in disease (9–11). It is now feasible to perform reliable high-throughput expression analysis of all known miRNA, currently 1426 in human (miRBase v17), across many experimental samples (12,13). As miRNA-mediated regulation is guided by sequence complementarity, several miRNA target prediction algorithms have been developed and used to generate miRNA target databases (8,14,15). Also, given that miRNA-directed binding of RNA-induced silencing complex may result in the degradation of the target mRNA, it is also possible to determine miRNA targets by examining significant inverse correlations between miRNA and mRNA expression data (16–18).

Several computational methods have been proposed that aim to integrate, to varying extents, sequence and expression information provided by the above sources to improve overall accuracy of miRNA target prediction. Ideally, correlations should be calculated between miRNA and mRNA expression data derived from the same sample (matched), but one may also measure correlations over sample classes in different data sets. Joung *et al.* applied a co-evolutionary learning approach to discover sets of co-expressed miRNA that had a maximal mean-predicted binding score to a set of co-expressed mRNA (19). However, the fitness function did not use any direct input from miRNA–mRNA expression correlations. Results were limited to the retrieval of two miRNA functional modules marginally enriched for three Gene Ontology (GO) categories. Liu *et al.* modelled miRNA–mRNA interactions as a directed bipartite graph and learned optimal Bayesian network structures constrained by miRNA target predictions (20). They used this approach to identify inversely expressed miRNA and mRNA across normal and cancer sample classes and identified miRNA functional modules comprising 6 miRNAs and 127 mRNAs that were related to the disease mechanism. Later, the same group developed an alternative probabilistic approach to model miRNA–mRNA interactions within expression data across a panel of mouse models (21). However, information on putative target prediction was not integrated with the expression data during the learning phase and was only used to evaluate discovered miRNA–mRNA modules. The rationale for this approach was to avoid bias from predicted targets; however, this need not be an all or nothing decision. While predicted target information may contain false positives and negatives (22), which may under- or over-constrain integrated methods, respectively, this information should not be wholly withheld from the learning phase. Rather, a more balanced approach should be pursued in which this information is included either with a reduced weight or at a later stage in the integration to aid fine-tuning of miRNA modules.

A pipeline for the integration of miRNA and mRNA data to model miRNA functional modules was developed by Sales *et al.* (23). This method facilitates the integration of both matched, via a correlation metric (Spearman’s rank, Pearson, mutual information), and unmatched miRNA and mRNA samples, using Chi-squared (or Pearson correlation 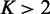), with predicted target information from several sources. Sales *et al.* incorporate the GenMiR++ Bayesian model, developed earlier (24,25), within a web-based analysis and visualization platform. GenMiR++ scores each miRNA according to how much it contributes to explaining the downregulation of an individual mRNA target, in the context of all other miRNAs that also target this mRNA. However, the approach does not model the interactions of miRNAs with multiple mRNA targets and as such fails to capture any modular structure within the miRNA–mRNA networks.

Huang *et al.* recently introduced the *MirConnX* tool for querying miRNA–mRNA interactions (26). *MirConnX* integrates a directed network of miRNA, mRNA and transcription factors, derived from predicted interactions, with an associative network derived from matched miRNA and mRNA expression data. The user then selects two thresholds, the ‘prior weight’ and the ‘regulation strength’, before being presented with a potentially large network of miRNA-mRNA and transcription factors (TFs) and a list of predicted interactions. Analysis of a glioblastoma case study resulted in a network containing 56 miRNAs, 29 TFs and 1180 mRNAs with 1851 predicted interactions. Although *MirConnX* integrates and orders the data in terms of the strength of predicted interactions, which makes it useful for low-level querying of individual miRNA–mRNA interactions, the output is still somewhat overwhelming and there is no attempt to further organize these interactions’ functional modules.

Earlier, work by Peng *et al.* looked at discovery of complete bipartite graphs within a binary association matrix of miRNA–mRNA derived from the integration of expression and target prediction data (27). Although promising, implementation and the scale of the miRNA modules discovered was limited, with most modules containing only 1, and at most 3, miRNA. Perhaps the use of a third-party maximal biclique algorithm (28), originally proposed for unipartite graphs and unadapted for the current problem domain, constrained results.

Our current understanding of miRNA regulation is expanding to consider networks of co-regulated miRNA, miRNA modules, which affect multiple functionally related mRNAs (29). Such a network would provide both a level of redundancy for robust output and a mechanism to fine-tune the expression of functionally overlapping groups of genes. Identifying subsets of miRNAs that interact with groups of related mRNAs may also provide an improved method of miRNA target prediction and miRNA functional annotation, as each putative interaction would be supported by several neighbouring connections and possibly a level of mRNA functional enrichment.

This miRNA–mRNA network may be depicted as a bipartite graph in which edges have both a direction and a weight. Previous studies have only focused on a narrow definition of such networks in which the miRNA influences the mRNA in a direct negative manner. We propose to extend this network model to also include indirect interactions.

Once we have defined a model, the next problem is how to detect the subsets of miRNAs and mRNAs that best fit this model. If we represent the interaction between all miRNAs and all mRNAs as a global bipartite graph, the problem then becomes one of detecting the sub-graph that best fits this model. To achieve this, we must define both a suitable scoring function, which captures the important aspects of our model, and develop an efficient method of retrieving these miRNA–mRNA sub-graphs. This problem is conceptually similar to that of bicluster analysis, which has been applied separately to both miRNA expression (30,31) and mRNA expression data (32). However, the question of how to visualize and interpret bicluster results, in a manner similar to the dendrogramatic representation of conventional cluster analysis, is still open.

In this work, we aim to first extend the current model of miRNA–mRNA interaction networks beyond those developed in previous studies, second to use bicluster analysis to improve on the methods of recovering such modular networks from integrated data sets and finally to develop and implement a method for both the visualization and querying of such networks.

## MATERIALS AND METHODS

### Expanding the model of the miRNA functional module

There are several lines of evidence that support a higher level organization of miRNAs into functional modules:

1. Individual miRNAs target many mRNAs. miRNAs have, on average, 277 conserved predicted targets in human (8).
2. The 3' UTR of an individual mRNA generally has many predicted miRNAs target sites. Human mRNAs are predicted to be targeted by an average of 10.6 miRNAs (8).
3. miRNAs are often located together on the genome, and neighbouring miRNAs are frequently co-expressed (33). Fifty-seven genomic clusters (miRNAs within 10 kb or the same host gene) are present in the human genome (34).
4. Approximately 50% of miRNAs are located in genomic clusters that are transcribed as a polycistronic pre-cursor that is then cleaved into individual pre-miRNA (35).
5. miRNAs are organized into families based on sequence similarity, see downloads at (16), some of which are also co-expressed and/or members of the same genomic cluster.
6. Interacting proteins tend to share more miRNA target-site types than random pairs (36).
7. Co-expressed miRNAs can regulate functionally related mRNAs (37).
8. Certain miRNA pairs may act in a cooperative manner to regulate gene functional modules (38,39).

**Figure 1.**
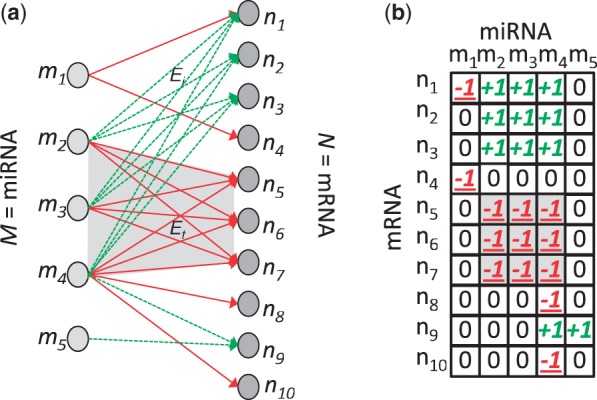
(**a**) A directed weighted graph bipartite *G*, with nodes representing a set of miRNAs, *M*, and set of mRNAs, *N*, and edges representing the set of interactions, *E*. Red arrows represent direct inversely correlated interactions, and green arrows represent indirect positively correlated interactions. The shaded area highlights the complete sub-graph or *biclique*, with edges *E_t_*, representing a group of miRNAs that post-transcriptionally downregulate a group of mRNAs. The dashed complete sub-graph, with green edges *E_i_*, represents a group of indirectly upregulated mRNAs (and positively correlated) to every miRNA in the direct biclique. (**b**) An idealized matrix of all miRNA-mRNA expression correlations across a matched set of samples. The values +1, −1 and 0 represent positive, negative and 0 correlations, respectively.

Typically, analysis of miRNA functional modules is confined to the assessment of post-transcriptional downregulation, as evidenced experimentally, by inversely correlated expression profiles, and computationally, by the presence of predicted 3' UTR binding sites. Using these data, a directed weighted bipartite graph, *G*, that represents the set of miRNAs, *M*, and the set of mRNAs *N* and their predicted interactions, *E*, may be constructed. It may also be informative to assess additional indirect interactions, particularly those of upregulated mRNAs that might better reflect the resultant phenotype and aid in the functional annotation of the miRNA module. These downstream interactions may also form an accompanying complete sub-graph, edges *E_i_* in Figure 1a. In this way, we can attempt to model the direct input and the downstream output of a set of miRNAs and assign a functional annotation of the miRNA module. Although this may provide an incomplete mechanistic model, akin to piecing together the corners and edges of a jigsaw puzzle, it may be used as a starting point from which to build a more complete understanding of the particular miRNA functional module.

In this study, we build our model on typical direct downregulating miRNA–mRNA interactions and indirect upregulating miRNA–mRNA interactions. This model is based on only two assumptions that miRNA–mRNA interactions depend on sequence-specific binding and that binding results in an inverse correlation of expression. Incorporation of further assumptions would provide additional constraints but also create conflicts and introduce errors that might ultimately hinder the unsupervised search for these complete sub-graphs or biclusters.

### Scoring and discovery of miRNA functional modules

**Figure 2.**
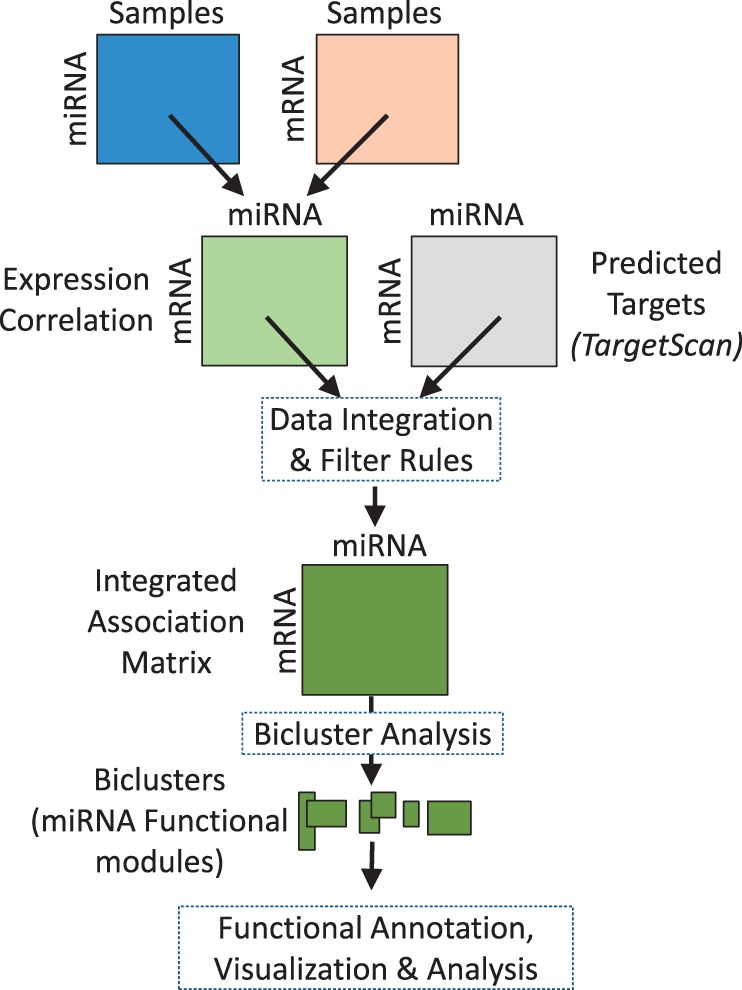
Overview of the analysis pipeline for miRNA functional module discovery consisting of data integration, bicluster analysis and miRNA module visualization.

The overall analysis pipeline for discovering miRNA–mRNA functional modules is presented in Figure 2. miRNA module definition and data integration are described in the following sub-section on ‘Data and Metrics’. Our case-specific extensions to the miRNA–mRNA discovery algorithm, based on bicluster analysis, are described in the subsection ‘miRNA module search algorithm’.

#### Data and metrics

First, we integrated miRNA and mRNA expression data sets and compiled a 2D correlation matrix for all possible combinations of miRNA and mRNA expression. In the idealized case, depicted in Figure 1b, the matrix may contain values +1, −1 and 0, representing positive, negative and zero correlations, respectively. In this case, a co-expressed miRNA module that downregulates a set of mRNA would be represented by a sub-matrix containing −1 at each element. Similarly, a downstream upregulated set of mRNAs would be represented by a sub-matrix containing +1 at each element.

Using this definition, a good solution within a correlation matrix derived from real miRNA and mRNA expression data would be represented by subset of miRNA (rows), *n*, and subset of mRNA (columns), *m*, with a low mean correlation, 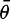. This solution may vary in both size (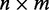) and quality (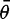), and given that we have some information to guide an approximation of the size of a typical miRNA module, i.e. average numbers of predicted targets per miRNA and *vice versa*, we may fix the area and minimize the correlation. We propose the following objective function to score selected sub-matrices *C*(*IJ*) within the expression correlation matrix *C*(*MN*):
(1)
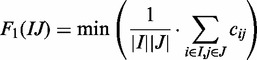

where *I* is the subset of rows and *J* is the subset of columns in the sub-matrix and *c_ij_* is the entry at row *i* and column *j*. We also have further interaction information, in the form of computationally predicted miRNA targets, that we can incorporate. For example, we may filter the correlation matrix to remove associations that are in conflict with predicted target site information. We suggest the following rules for filtering miRNA–mRNA (*MN*) expression correlations (*C*) based on direction of correlation (negative or positive) and presence or absence of predicted target site (*T*) to produce an integrated association matrix (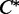) as follows:

- if (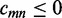 AND *t_mn_* = TRUE) then 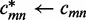
- if (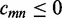 AND *t_mn_* = FALSE) then 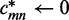
- if (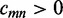 AND *t_mn_* = FALSE) then 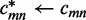
- if (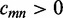 AND *t_mn_* = TRUE) then 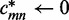

This method of data integration acts to neutralize conflicting information from the two data types producing a cleaner data set before cluster analysis, or in this case, a smoother search space for the ensuing bicluster search algorithm. By assigning a value of 0 where a conflict exists, we neutralize its impact on the objective function described above. Now that we can assess the quality of a potential miRNA functional module within the integrated association matrix, we now require a suitable method of searching the solution space in a thorough and efficient manner.

#### miRNA module search algorithm

The procedure of locating an optimal subset of rows and columns within a data matrix that satisfies a selected criterion is akin to simultaneous two-way clustering or ‘biclustering’ of rows and columns. The number of possible solutions increases exponentially with the size of the data matrix and quickly becomes unsolvable by exhaustive means. Various metrics and optimization algorithms have been proposed to bicluster large gene expression data sets to model gene functional modules (32,40,41). The BUBBLE biclustering algorithm uses simulated annealing (SA) search method, which allows it to by-pass local minima, to locate highly correlated bicluster ‘seeds’ within a gene expression data matrix. These are then expanded in a deterministic manner by addition of the correlated rows and columns (42).

In the current context, we can apply a similar approach incorporating the objective function in Equation 1, to locate the high-scoring ‘seeds’ within an integrated miRNA–mRNA association matrix. The objective function is first applied to locate seeds composed of a set of miRNAs that are directly inversely correlated to a set of mRNAs, i.e. sub-matrices within the integrated miRNA–mRNA association matrix composed of a maximum number of negative values (see the shaded ‘biclique’ and sub-matrix in Figure 1). We may then deterministically expand these seeds by the sequential addition of columns (miRNA) and rows (mRNA), with maximum mean inverse correlation relative to the seed. We selected a column threshold of 10 and a row threshold of 250; these values approximate the mean number of miRNAs predicted to target each mRNA and the mean number of mRNA predicted to be targeted by each miRNA, see ‘Expanding the model of the miRNA functional module’ section. In the case of rows (mRNA), we may also add those that have a maximum mean positive correlation to reflect mRNAs that are coordinately upregulated downstream and which may aid characterization of the resultant phenotype of the miRNA functional module. For further details see (41,42) and Supplementary Methods.

## miRNA–mRNA MODULE VISUALIZATION

In this section, we present our novel bicluster visualization method, as applied to miRNA–mRNA functional modules. We then describe the implementation of this method within a practical visualization and analysis software application.

### Bicluster visualization

Standard cluster analysis may be visualized in several ways, with perhaps the most common being a hierarchical dendrogram. However, the problem of bicluster visualization is far from straight forward. The issues of calculating object and cluster similarity over differing, even exclusive, feature subsets and the prospect of objects belonging to more than one bicluster complicate visualization efforts. Santamaría *et al.* attempt to visualize gene expression biclusters as a force-directed graph where bicluster similarity is based on the degree of gene and condition overlap (43). We find that attempting to directly depict each shared object (mRNA or miRNA in our context) produces a rather cumbersome bicluster visualization, defeating the objective somewhat.

We have developed a visualization method that renders a set of biclusters as a 2D bubble plot. In this context, the *X*-axis represents bicluster similarity based on the extent of miRNA overlap, and the *Y*-axis represents bicluster similarity based on mRNA overlap. Both cases involve calculating the similarity of two lists of labels, i.e. miRNA or mRNA labels. We use the following metric for measuring the similarity between two lists *L*_1_ and *L*_2_:
(2)
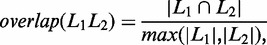



This overlap metric avoids biases due to bicluster size (that would be incurred if one were to simply use 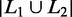 as the denominator) and also obeys the ‘triangle inequality’ rule, i.e. 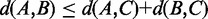, allowing the similarity of a set of biclusters to be more accurately projected relative to one another onto one or more plotting dimensions.

Using this *overlap* metric, two similarity matrices [range (0,^1^)], one representing miRNA similarity and the other mRNA similarity, may be calculated between all biclusters. Classic multidimensional scaling (MDS) [see (44)] is then used to project the miRNA and mRNA similarity matrices onto the *X-* and *Y-*axes, respectively.

**Figure 3.**
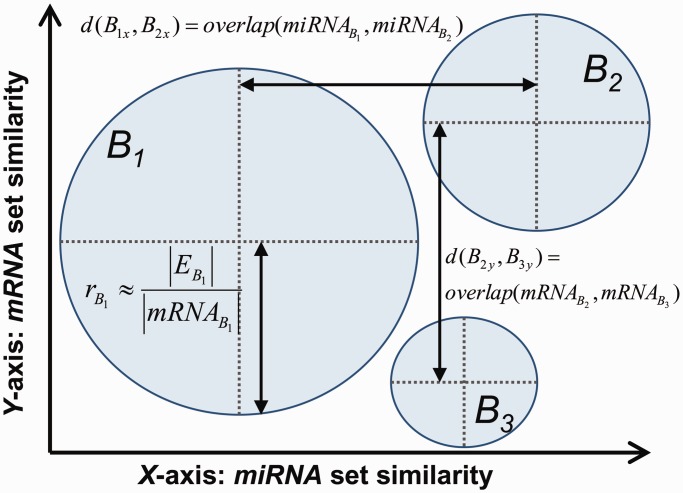
Visualization of biclusters representing miRNA–mRNA functional modules as a 2D bubble chart. The similarities in terms of miRNA overlap and mRNA overlap are projected on the *X-* and *Y-*axes, respectively. The radius (*r*) of each ‘bubble’ is proportional to the size (as a percentage of the mRNAs in the bicluster) of the most enriched mRNA functional category (*E*) for that miRNA–mRNA module.

The size of a point or ‘bubble’ in the plot representing an miRNA–mRNA functional module is proportional to the size of the most enriched functional category (as a percentage of the overall bicluster size), i.e. a larger bubble has a greater coherence with its most enriched functional category (see Figure 3). In this study, we use Gene Ontology (GO) enrichment information, based on the mRNAs within the miRNA–mRNA functional module, to assign a functional category. We also assess miRNA functional modules in terms of enrichment for known miRNA families and genomic clusters.

### miRNA–mRNA module visualization software

**Figure 4.**
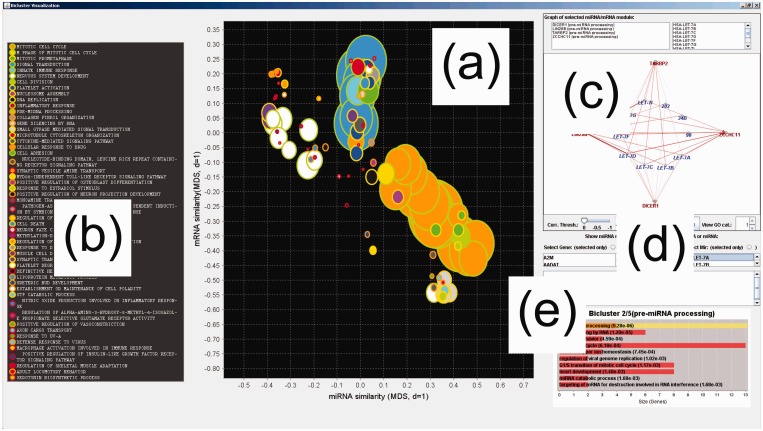
(**a**) Interface of the custom software application built to support visualization and (**b**) functional annotation of all miRNA modules (biclusters) uncovered in a data set. (**c**) Detailed viewing of the members and interactions within individual miRNA functional modules, (**d**) querying of an individual miRNA or mRNA of interest and (**e**) detailed GO enrichment analysis based on the mRNAs within the module is also supported.

We have implemented our method of miRNA functional module visualization within an interactive Java application, ‘miRMAP’, that allows the user to visualize and query selected modules and individual miRNAs or mRNAs (see Figure 4). Different GO categories are rendered as bubbles with a unique combination of perimeter and area colours; therefore, a modest palette of 10 colours can render up to 100 distinct GO categories. It is also necessary to order the modules on the *Z*-axis by size to allow all modules to be visible, i.e. smaller modules overlaid on larger modules.

Functional modules within the bubble plot may be selected by left clicking on the bubble interest, and plotted in the neighbouring panel as a bipartite graph presented in a compact radial layout. Inversely associated miRNA–mRNA are connected via red edges and positively miRNA–mRNA associations are represented as green edges. Correlation thresholds can be dynamically adjusted to fine-tune the visualization of nodes and edges in each miRNA module. Functions are assigned to each miRNA module using the most over-represented GO category within the mRNA present in the module. The top 10 significant GO categories are also shown in the bottom right panel as a bar chart with associated *P*-values based on the hypergeometric distribution. A subset of nodes and interactions within a module, pertaining to any one of these functional modules, may also be viewed. Finally, modules containing an mRNA or miRNA of interest may be selected and viewed. A Java implementation of the full analysis and visualization pipeline, or miR Module Analysis Program (miRMAP), is available at https://www.dropbox.com/s/pnluebfq9lto6iw/miRMAP.zip.

### Data set description

#### Neuroblastoma

Neuroblastoma is a paediatric cancer arising from precursor cells of the sympathetic nervous system. It has several genetic subtypes that range in outcome from spontaneous regression to rapid progression and death. There are several markers and genomic imbalances that correlate with subtype and survival, the most notable of which is amplification of the *MYCN* oncogene. The data consist of 42 matched neuroblastoma tumour samples for which both miRNA and mRNA expression profiling was carried out. The miRNA expression data consist of 298 miRNA, and was reported previously as part of a larger study (12). The mRNA expression profiling was carried out with GeneChip Human Exon (1.0 ST) Array, which contains 22 011 core transcripts.

#### Human immune cell subsets

The second data set is derived from an miRNA and mRNA expression profiling of human immune cells (45). Nine cell subsets (CD16+CD66b+ neutrophils, CD16-CD66b+ eosinophils, CD14+ monocytes, CD4+ T cells, CD8+ T cells, CD56+ NK cells, CD19+ B cells, CD123+ pDCs and CD11c+ mDCs) were profiled for mRNA, using the Affymetrix U133 Plus 2.0 chip, and miRNA, using the Affymetrix miRNA chip.

## RESULTS

### Pre-processing the association matrix

First, mRNA–miRNA expression correlation matrices for each data set were compiled using Spearman’s rank correlation. This matrix was then integrated with predicted miRNA target data from TargetScan V5.1 using the rules defined in ‘Data and metrics’ section. It should be noted that no correlation threshold was enforced at this stage, i.e. it was sufficient to have no conflict between the expression and predicted targeting of an miRNA for the value to be included in the association matrix; see Supplementary Data for further details. After this compilation and filtering, the neuroblastoma miRNA-mRNA integrated association matrix contained 13 122 mRNAs and 201 miRNAs. The human immune cell miRNA-miRNA integrated association matrix contained 15 735 mRNAs and 677 miRNAs.

### Cooperativity within miRNA clusters and families

Previous studies have used the term ‘synergy’, in the context of miRNA regulation, to describe pairs of miRNAs that significantly co-regulate at least one functional module (39). Another study, examining the effects of multiple miRNAs binding to a single mRNA, discriminates between types of cooperativity, where the result of co-targeting is equal to the sum of the individual effects, ‘additive’, or greater, ‘synergistic’ (38).

Here we examine whether one or both types of targeting cooperativity can be computationally detected between members of the same miRNA family or genomic cluster. miRNA family information is available at (16) and genomic clusters where generated using (34) (selecting a 10 kb or the same gene as a window) and are given in Supplementary Table S1. Should a level of cooperativity be present for a given miRNA family/cluster, one would expect the effect on mRNAs, as measured by level of inverse expression, to increase if they are targeted by more members of a miRNA cluster/family.

**Figure 5.**
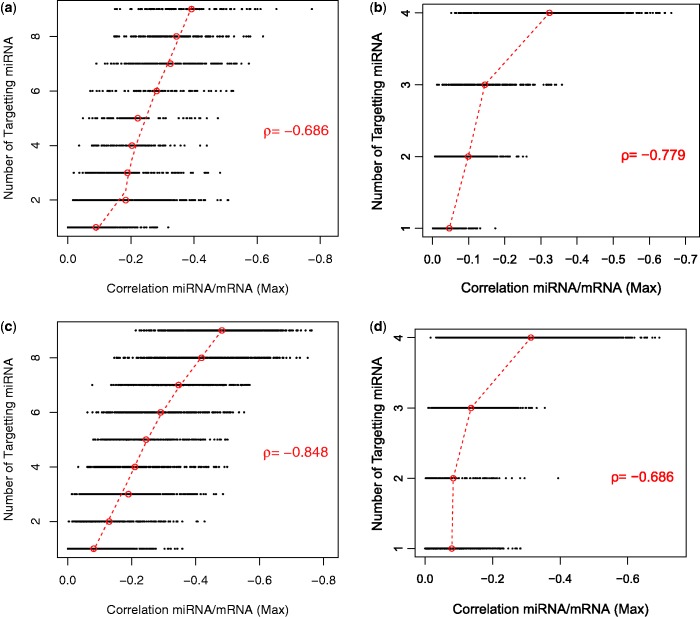
miRNA cooperativity. Scatter plots showing the number of miRNAs targeting an mRNA (*Y axis*) versus the correlation between miRNAs and mRNA expression (*Y axis*) for selected miRNA families within the integrated data set for neuroblastoma (**a**) the let-7 family and (**b**) and mir-302 family, and human immune cells (**c**) the let-7 family and (**d**) the mir-320 family. A point in a plot represents the maximum (Max) correlation for a miRNA family and a mRNA target. The level cooperativity is calculated using Spearman’s rank correlation (ρ) of the median points (circled in red) for each level on the *Y* axis. See Table 1 for significance values.

We have examined mRNA groups that are targeted between 1:*N* times for each miRNA cluster/family, where *N* is the size of the miRNA cluster/family. Where there is more than one miRNA targeting an mRNA, the maximum inverse correlation for these interactions is taken. Scatter plots of the number of targeting miRNA versus miRNA–mRNA expression correlations for selected miRNA families/clusters, from both data sets, are shown in Figure 5. Each point in the scatter plot represents the correlation of one mRNA target (*X*-axis) and one or more miRNAs (*Y*-axis). In some cases, we can see a clear trend as the median inverse correlation of the points increases in magnitude as the number of miRNA from the family/cluster targeting these mRNA increases (see Figure 5). This trend or association with cooperativity is measured by Spearman’s rank correlation (ρ). Significance for each miRNA family/cluster is assessed based on a background of 100 randomly generated miRNA groupings of the same size (see Table 1 and Supplementary Table S2). Existence of significant cooperativity, across both data sets, for genomic clusters up to 32 miRNAs in size and families up to 23 miRNAs adds support for the presence of coordinated interplay within miRNA families and clusters.

**Table 1.** miRNA clusters/families (*n* > 2) that exhibit significant cooperativity in the integrated Neuroblastoma or Human Immune data sets

	Neuroblastoma			Human immune cells		
miRNA Cluster/Family	*n*	Cooperativity (ρ)	*P*	*n*	Cooperativity (ρ)	*P*
let-7A-1 cluster	3	−0.73	< 0.01	3	−0.73	< 0.01
mir-106A cluster	4	−0.46	0.01	6	−0.48	< 0.01
mir-106B cluster	3	−0.35	< 0.01	3	−0.42	0.02
mir-1185 cluster	20	−0.48	0.17	32	−0.58	< 0.01
mir-127 cluster	4	−0.26	0.3	7	−0.35	0.01
mir-1283 cluster	6	−0.44	0.01	27	−0.56	< 0.01
mir-17 cluster	4	−0.22	0.6	6	−0.51	< 0.01
mir-181C cluster	4	−0.47	0.01	5	−0.36	< 0.01
mir-216A cluster				3	−0.45	< 0.01
mir-23B cluster	3	−0.31	0.03	3	−0.23	0.81
mir-302A cluster	5	−0.42	0.02	5	−0.39	0.03
mir-424 cluster				3	−0.36	< 0.01
mir-506 cluster				6	−0.38	< 0.01
let-7 family	9	−0.69	< 0.01	9	−0.85	< 0.01
mir-130 family				4	−0.69	< 0.01
mir-148 family	3	−0.37	< 0.01	3	−0.78	< 0.01
mir-15 family	4	−0.62	< 0.01	4	−0.66	< 0.01
mir-154 family	12	−0.39	0.45	15	−0.49	< 0.01
mir-17 family	6	−0.60	0.01	8	−0.75	< 0.01
mir-181 family	3	−0.56	< 0.01	4	−0.71	< 0.01
mir-29 family	3	−0.71	< 0.01	3	−0.76	< 0.01
mir-30 family	3	−0.73	< 0.01	5	−0.84	< 0.01
mir-302 family	4	−0.78	< 0.01	5	−0.35	0.16
mir-320 family				4	−0.69	< 0.01
mir-329 family				3	−0.34	0.04
mir-368 family				3	−0.25	0.02
mir-379 family				5	−0.28	< 0.01
mir-506 family				7	−0.36	< 0.01
mir-515 family	4	−0.29	0.14	23	−0.52	< 0.01
mir-548 family				14	−0.50	< 0.01
mir-743 family				4	−0.30	0.01
mir-8 family	4	−0.32	0.08	5	−0.27	< 0.01
mir-95 family				4	−0.28	0.03

Association with cooperativity of number of targeting miRNA versus inverse expression, is calculated using Spearman’s Rank Correlation (ρ). Significance (*P*) is calculated based on a background of 100 randomly generated miRNA groupings for each miRNA cluster/family size(*n*). See Supplementary Table S2 for full set of results.

We also note that some miRNA groupings, such as the hsa-let-7 family, show significant cooperativity across both data sets (see Table 1 and Figure 5a and c). Cooperativity within miRNA families/clusters seems to be chiefly additive. In some cases, there is limited evidence of a greater effect, such as in the plots of the mir-302 family in the neuroblastoma data set (Figure 5b) and the mir-320 family in the human immune cells data set (Figure 5d). A further more comprehensive analysis of this phenomenon is warranted but is outside the focus of this current study.

In the next section, we examine whether the mRNAs targeted by such miRNA modules have significant functional coherence. We also consider that the notion of a miRNA functional module may not directly correspond to known miRNA genomic clusters or families, i.e. being made up by subsets of one or more cluster/family or an entirely unknown grouping miRNAs. As such, we have used a wholly unsupervised method in the next section, which seeks out coherent patterns within the integrated data sets without referencing any miRNA or mRNA class information. Such information is only used *post hoc* for module functional annotation and evaluation.

### Unsupervised modelling and analysis of miRNA functional modules

In this section, we first retrieve miRNA–mRNA functional modules from the integrated association matrix in a wholly unsupervised manner by using a bicluster analysis approach using the BUBBLE algorithm (41) modified for the current context. Results are visualized and interpreted using a novel visualization and analysis application, ‘miRMAP’, described in the ‘miRNA-mRNA Module Visualization Software’ section and shown in Figure 4. Here we evaluate the discovered miRNA–mRNA functional modules in terms of GO category and miRNA cluster/family enrichment and assess agreement with both known, experimentally validated miRNA–mRNA target pairs and our own miRNA transfection data.

#### Neuroblastoma tumour data

**Figure 6.**
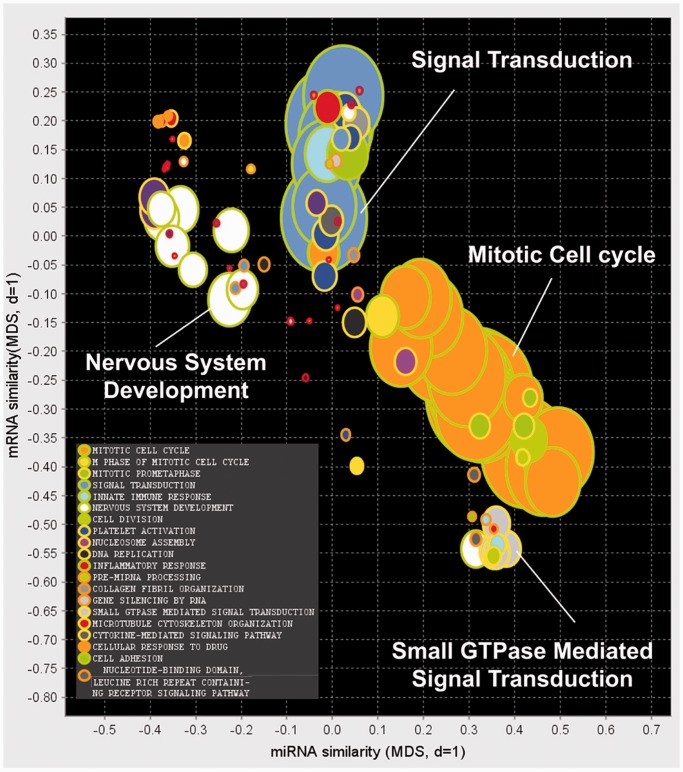
Visualization of miRNA modules uncovered in the neuroblastoma tumour data set. The miRNA similarity and mRNA similarity of the miRNA modules (see Equation 2) are projected onto the *X-* and *Y-* axes, respectively, using classic multidimensional scaling. The colour and diameter of a bubble in the plot represent the type and size, respectively, of the most significantly enriched GO category in the miRNA–mRNA functional module. miRNA functional modules annotated with the functions ‘Mitotic Cell Cycle’, ‘Nervous System Development’, ‘Signal Transduction’ and ‘Small GTPase-Mediated Signal Transduction’ make up one-third of the biclusters discovered.

Bicluster analysis uncovered 100 putative miRNA functional modules from the integrated association miRNA–mRNA matrix derived from neuroblastoma data. Functional labels were assigned to each miRNA module depending on the most significantly enriched GO category within the associated mRNAs. The full set of miRNA modules was visualized using our custom software application, outlined in Figure 4, and presented in Figure 6. The most frequent categories that occurred in this data set were Mitotic Cell Cycle:18 modules (orange, green border), Nervous System Development:9 modules (white, green border), Signal Transduction:6 modules (blue, green border) and Small GTPase Mediated Signal Transduction:4 modules (light gray, yellow border). The similarity of these miRNA functional modules in terms of miRNA and mRNA membership is reflected in their position on the X- and Y-axes, respectively. For example, we can see from the X-axis separation of the three most frequent modules that they differ their miRNA membership.

The most significant miRNA modules for each functional category are shown in Table 4 and full results are given in Supplementary Table S3. Here we also present the most significantly over-represented miRNA family/cluster for each miRNA functional module.

**Figure 7.**
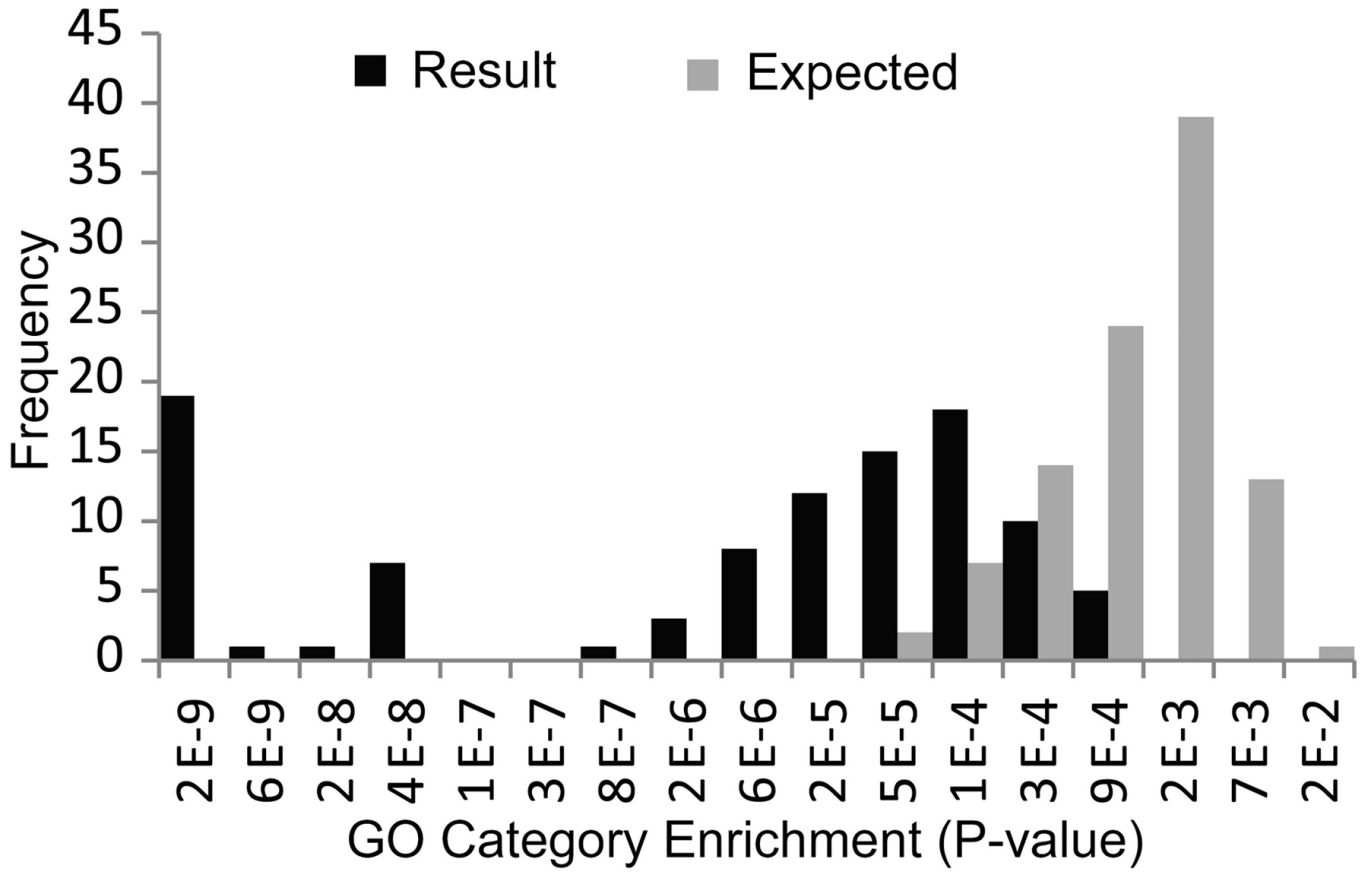
The spread of significance values (based on Gene Ontology functional enrichment) of the 100 miRNA modules retrieved from the neuroblastoma data set (black bars) compared with the expected distribution (grey bars), i.e. 100 biclusters generated after mRNA labels were randomly permuted. miRNA family/cluster enrichment for these sets is examined in Table 2.

To address the question of the likelihood of retrieving such a set by chance given the null hypothesis (i.e. if there were no miRNA–mRNA functional modules within the data set), we also retrieved 100 miRNA modules after miRNA and mRNA labels had been randomly permuted. Figure 7 displays the distribution of GO significance values for each of these results. A significant (*P* < 4.43e-13) difference in the distributions is observed. The result contains 62 miRNA modules that are more significantly enriched (many far more so as can be seen in the distribution) for GO categories than any of the 100 found by chance. We also see that our result contains significantly (*P* < 1e-5) more miRNA modules with miRNA family/cluster over-representation (*P* < 0.05) than one would expect by chance, see Table 2.

**Table 2.** miRNA functions module miRNA family/cluster enrichment

	Result	Expected
miRNA cluster		
Significantly enriched	73	8
Non-significant	27	92
miRNA family		
Significantly enriched	78	19
Non-significant	22	81
	Chi-square: *P* < 1e-5	

The number miRNA functional modules that are significantly enriched for miRNA clusters and families are significantly more (Chi-squared *P* < 1e-05) than expected (found by chance in the randomly permuted data set).

Finally, we also evaluate the overall result by examining agreement with 1093 experimentally validated miRNA–mRNA target pairs in human, from *Tarbase V5* (46), see Table 3 and Supplementary Table S5. The overall set of 100 miRNA modules is supported by 95 experimentally validated miRNA–mRNA target pairs, as compared with only 5 as expected by chance. After standardization for total coverage of miRNA–mRNA target pairs, the density of experimentally validated miRNA–mRNA target pairs is still 14.3-fold more than expected by chance. We now examine some selected miRNA modules in more detail.

**Table 3.** Evaluation of miRNA functional modules discovered in the neuroblastoma data set with experimentally validated miRNA–mRNA target pairs, see Supplementary Table S5 for full set of results

Evaluation Criterion	Result	Expected
Total miRNA modules retrieved	100	100
miRNA modules with ≥1 validated miRNA-target pairs	38	4
Mean number of miRNAs in miRNA modules	19.8	14.9
Mean number of mRNAs in miRNA modules	235.5	236.7
Total validated miRNA-target pairs in miRNA modules	95	5
Density of validated miRNA-target pairs in miRNA modules	0.00078	5.4e-05

**Figure 8.**
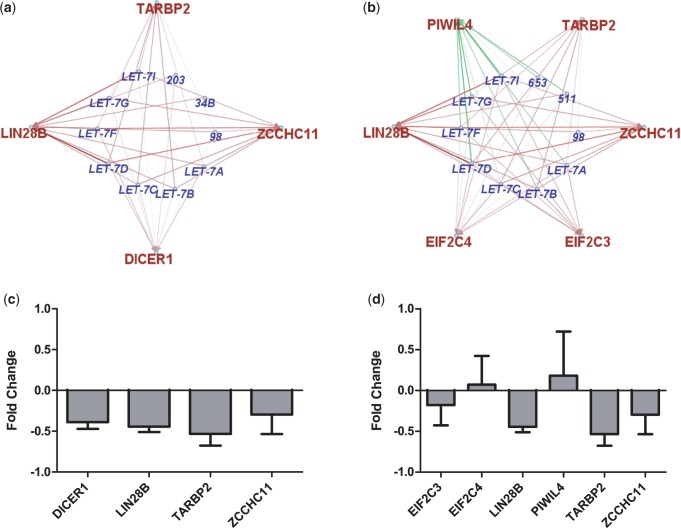
(**a**) ‘miRNA processing’ and (**b**) ‘gene silencing by RNA’ functional modules found in the neuroblastoma tumour-integrated data set. These hsa-let-7a interactions are supported by hsa-let-7a transfection results shown in (**c**) and (**d**), respectively. miRNA (outer green node) and mRNA (inner red node) are connected via red (inversely correlated) and green (positively correlated) edges, the thickness of which is proportional to the magnitude of correlation.

The top 20 miRNA modules, in terms of GO enrichment, are given in Table 4, and all have a GO enrichment more significant than any found by chance in the randomly permuted data set. Selected miRNA functional modules, involving the let-7 family interaction with ‘pre-miRNA processing’ and ‘Gene silencing by RNA’, are shown in further detail in Figure 8. We also present data from a transfection of a member of this module, hsa-let-7a, in the neuroblastoma cell line KELLY, that adds support to the mRNA nodes and relevant edges in this module. All mRNA members of ‘pre-miRNA processing’ miRNA module are downregulated as predicted after transfection (see Figure 8a and c). Some support can also be seen for the ‘Gene silencing by RNA’ miRNA module where, following transfection of hsa-let-7a, EIF2C3, LIN28B, TARBP2 and ZCCHC11 are downregulated and PIWIL4 is upregulated.

**Table 4.** The top 20 miRNA-mRNA functional modules, in terms of enrichment for Gene Ontology (GO) categories, for the neuroblastoma integrated data set returned by biclustering

GO category	n	S	*P*-value	miRNA cluster/family	n	N	*P*-value
Mitotic cell cycle	57	250	2.0e-39	mir-17 cluster	3	6	3.0e-03
M phase of mitotic cell cycle	18	220	2.0e-15	mir-8 family	2	5	2.1e-02
Mitotic prometaphase	11	154	5.9e-10	mir-17 family	5	8	7.3e-05
Signal transduction	48	250	9.6e-10	mir-200a cluster	2	3	1.1e-02
Innate immune response	24	250	3.7e-08				
Nervous system development	23	250	5.9e-08	mir-135 family	2	2	6.5e-03
Cell division	19	250	7.0e-08	mir-17 family	6	8	7.6e-06
Platelet activation	16	250	8.4e-08	mir-29 family	3	3	7.9e-04
Nucleosome assembly	12	250	9.0e-08	mir-181 family	2	4	1.5e-02
DNA replication	13	213	9.6e-08	mir-192 cluster	2	2	1.0e-02
Inflammatory response	15	250	7.3e-06	mir-489 cluster	2	2	6.5e-03
pre-miRNA processing	4	250	9.3e-06	let-7 family	8	9	1.4e-07
Gene silencing by RNA	6	250	1.2e-05	let-7 family	8	9	1.4e-07
Collagen fibril organization	6	250	1.2e-05	mir-15 family	4	4	1.1e-04
Small GTPase-mediated signal transduction	16	250	1.9e-05	mir-302a cluster	5	5	2.0e-03
Microtubule cytoskeleton organization	7	250	2.1e-05	mir-329 family	2	3	1.1e-02
Cytokine-mediated signaling pathway	13	250	2.0e-05	mir-8 family	3	5	2.1e-03
Cellular response to drug	5	250	2.6e-05	mir-329 family	2	3	1.1e-02
Cell adhesion	22	250	3.0e-05	let-7 family	7	9	1.3e-06
Nucleotide-binding domain, leucine rich repeat containing receptor signaling pathway	6	250	4.1e-05	mir-302a cluster	5	5	6.8e-03

Enrichment for miRNA genomic clusters and families in human is also shown were applicable. The number of members found in a category (*n*), the number total of members in the category (*N*) and the total number of mRNA (*S*) are given. See Supplementary Table S3 for full set of results.

Several miRNA and mRNA target genes have been implicated in neuroblastoma pathogenesis (47). In some cases, miRNAs have been identified but a specific target and mechanism has yet to be elucidated. For example, it is known that the endogenous knockdown of hsa-miR-7 promotes neurite outgrowth in neuroblastoma cell lines, but no target or mechanism has been identified. Similarly, hsa-miR-214 has been shown to promote neurite growth when ectopically over-expressed, but a specific target and mechanism remain elusive (48).

**Figure 9.**
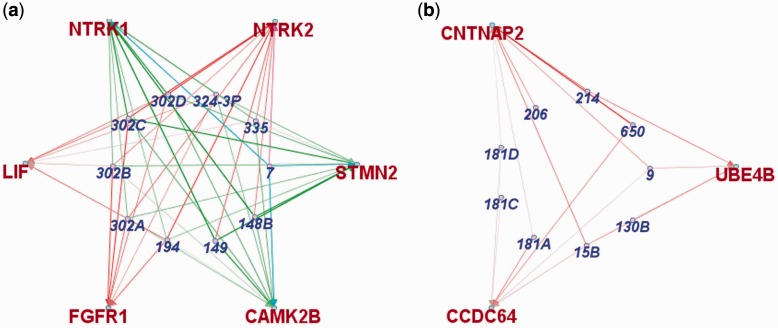
(**a**) miRNA module containing hsa-mir-7 enriched for the GO category ‘positive regulation of neuron projection development’ (**b**) miRNA module containing hsa-mir-214 enriched for the GO category ‘neuron projection development’.

Using our visualization and analysis software application, the modular miRNA-mRNA network revealed by our approach may be queried for individual miRNA or mRNA relationships. In Figure 9a, we see that hsa-miR-7 is a well-connected node within the module ‘Positive Regulation of Neuron Projection Development’ that is principally enriched for the mir-302a family/cluster (*P* < 1.9-04). The connection between hsa-miR-214 and neurite growth was not evident in the miRNA modules uncovered in the initial unsupervised analysis. However, guided by the phenotype and supported by our custom software application, further interrogation of hsa-miR-214 containing modules, primarily enriched for ‘Mitotic Cell Cycle’, yielded a sub-module module enriched for ‘Neuron Projection Development’ shown in Figure 9b.

#### Immune cell data

**Figure 10.**
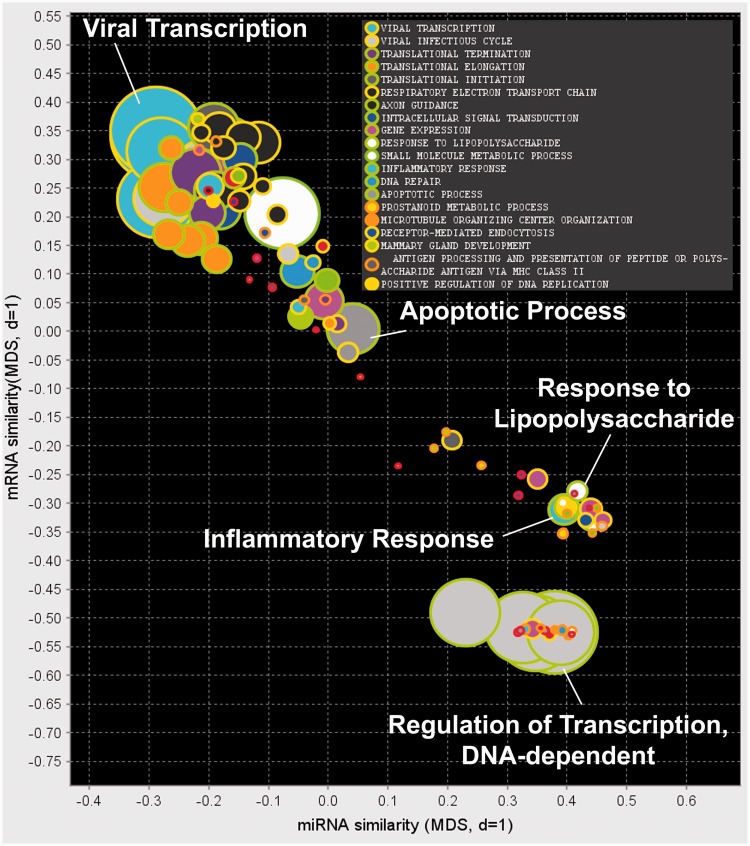
Visualization of miRNA modules in immunology data set.

To assess the broader application of our method of modelling miRNA functional modules, we also applied this technique to a publicly available data set differing in cell type; see the section Dataset Description and (45). Results of the unsupervised bicluster analysis are visualized in Figure 10, and the top 20 miRNA-mRNA functional modules for each category are presented in Table 5 and Supplementary Table S4.

**Table 5.** The top 20 miRNA functional modules, in terms of enrichment for GO categories, uncovered in the Human Immune Cells data set

GO category	n	S	*P*-value	miRNA cluster/family	n	N	*P*-value
Viral transcription	39	198	5.1e-45	mir-320 family	4	4	1.1e-04
Viral infectious cycle	28	202	2.0e-28	mir-146 family	2	2	6.5e-03
Translational termination	21	199	3.4e-20	mir-181a cluster	2	2	5.5e-03
Translational elongation	21	198	1.2e-19	mir-320 family	4	4	1.1e-04
Translational initiation	21	196	1.1e-17	mir-320 family	4	4	1.1e-04
Respiratory electron transport chain	18	220	6.4e-15	mir-181a cluster	2	2	6.5e-03
Intracellular signal transduction	17	176	3.2e-08				
Axon guidance	17	176	3.2e-08	mir-320 family	4	4	1.1e-02
Gene expression	25	221	4.3e-07	mir-181a cluster	2	2	6.5e-03
Response to lipopolysaccharide	10	170	9.3e-07	mir-15 family	3	4	1.3e-03
Small molecule metabolic process	31	211	4.2e-06	mir-188 cluster	4	7	4.6e-04
Inflammatory response	13	182	5.0e-06	mir-17 family	4	8	6.6e-04
DNA repair	14	203	7.4e-06				
Apoptotic process	22	214	8.4e-06	let-7 family	9	9	1.5e-08
Prostanoid metabolic process	4	180	1.4e-05	mir-15 family	3	4	1.3e-03
Microtubule organizing center organization	3	158	2.0e-05	mir-17 family	8	8	6.8e-04
Receptor-mediated endocytosis	6	192	2.2e-05	mir-17 family	5	8	7.3e-05
Mammary gland development	5	199	2.5e-05	mir-320 family	4	4	1.1e-04
Antigen processing and presentation of peptide or polysaccharide antigen via MHC class II	4	162	3.0e-05	mir-500 family	2	3	2.1e-02
Positive regulation of DNA replication	5	175	4.5e-05				

Enrichment for miRNA genomic clusters and families is also shown were applicable. The number of members found in a category (*n*) and the number of members in the category (*N*) are given. See Supplementary Table S4 for full set of results.

**Figure 11.**
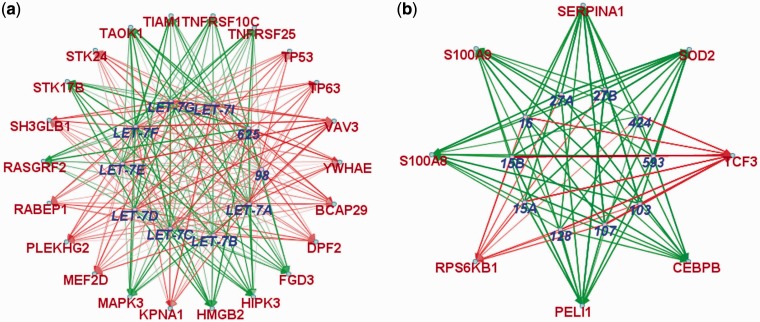
Selected miRNA modules found in the immune cells data set enriched for (**a**) the let-7 family and ‘Apoptotic process’ and (**b**) the mir-15 family and ‘response to lipopolysaccharide’.

Interestingly, we can see that again the let-7 family module is prominent in terms of miRNA family enrichment (*P* = 1.5E-08), with all 9 members occurring in the module related to ‘Apoptotic process’ (*P* = 8.4e-06); see Figure 11a. Another module that may be involved in the immune response is enriched genes related to the ‘Response to lipopolysaccharide’ (*P* = 9.3e-07) and for members of the mir-15 family (*P* = 1.3e-03), see Figure 11b.

Another prominent module, Viral Infectious Cycle (*P* = 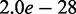), has enrichment for hsa-mir-146a family. It has been reported previously that mir-146a family is stimulated by viral infection in an NFκB related manner (49). Also prominent is the hsa-mir-181a cluster, which may be involved in haematopoietic cell lineage differentiation (50).

## DISCUSSION

As with previous trends in mRNA expression analysis, the elucidation of miRNA function may be aided by a systems-level approach. Accepting the premise that mRNAs may be co-regulated to perform specific functions, it seems natural that miRNAs may contribute to this modular regulation at a post-transcriptional level. Here we first examined the possible cooperativity of miRNA classes and saw that, where present, cooperativity generally appears to be additive in nature, having a linear relationship between the number of targeting miRNAs and the effect on target mRNAs. Some miRNA classes, such as the mir-302 family in the Neuroblastoma data set and mir-320 family in the Human Immune Cells data set appeared to show some limited evidence of synergistic cooperativity that may warrant further investigation. Interestingly, although the mir-302 family showed cooperativity in the Neuroblastoma data set, it did not show any significant cooperativity in the Human Immune Cells data set. It is intriguing to speculate that cooperativity itself may be modulated, e.g. by miRNA concentrations or other factors, to regulate the effect of miRNA module. Significant miRNA cooperativity for a number of miRNA clusters and families was also present across both data sets, despite differing cell types. It is of interest to note that certain miRNA classes, such as the hsa-let-7 and hsa-mir-29 families, showed significant cooperativity across data sets, perhaps due their regulation of fundamental cellular functions.

Supported in part by these findings, we investigated the functional basis for such cooperativity by applying a bicluster analysis to locate subsets of miRNAs that were associated with groups of mRNAs within integrated miRNA-mRNA association matrices. This unsupervised analysis uncovered significant evidence of an extensive modular miRNA–mRNA network by locating many modules, enriched both for specific miRNA cluster/families and GO Biological Process, in both data sets examined. It was also of interest to see that, although sparse in absolute terms, the coverage of experimentally validated miRNA-mRNA target pairs was 14.3-fold more than discovered by chance. In the Neuroblastoma data set, one-third of modules uncovered fell into the three categories—Cell Cycle, Signal Transduction and Nervous System Development—and reflected the dominance of these modules. Perhaps, somewhat expectedly, given both their prominence in the literature, the most significant evidence for modular miRNA regulation was provided by the hsa-let-7 family (*P* = 1.4e-07) and the hsa-mir-17 family (*P* = 7.6e-06). The magnitude and direction of influence of several edges within two hsa-let-7 functional modules were supported by hsa-let-7a cell line transfection data. The agreement across tumour and cell lines supports these targets and functions and again points to fundamental cellular functions of the hsa-let-7a family. Modules providing insight into the possible mechanism of how hsa-mir-7 and hsa-mir-214 regulate neuron projections in neuroblastoma were also uncovered, demonstrating how this approach may aid filling in the gaps in functional mechanism for specific miRNAs.

We also saw potential for miRNA functional module analysis to apply to other cell types with retrieval of an extensive modular network within the Human Immune Cell data set. Several miRNA modules with enrichment of immune-related functions, such as ‘Viral Transcription’, ‘Response to Lipopolysaccharide’ and ‘Apoptotic Process’, were uncovered. The ‘Apoptotic Process’ module is enriched for hsa-let-7 family members, which are found to be downregulated in several cancers, and may offer some mechanistic insight into their dysregulation. miRNA functional modules would meet the needs of cellular functions that require a rapid coordinated regulation, such as those involved in the immune response. Although this data set represented a normal unchallenged model, several immune-related functions are evident and we see potential for the future application miRNA functional module analysis in this area.

As we learn more about the transcriptional regulation and activity of miRNAs, we see potential to improve the modelling of miRNA functional modules and the accuracy of integrated miRNA-mRNA association matrices. The modelling, visualization and analysis methods developed here will still apply to such association matrices and can also be applied to other domains where bicluster analysis is used, such as high-dimensional gene expression or metabolomics data sets.

The question of how to visualize biclustering results is still open, and currently there is no generally accepted visualization method akin to the dendrogram used for cluster analysis. Although the software visually depicts similarity of miRNA functional modules, one way in which this approach might be further refined is by the implementation of a ‘merging’ step (either at the search or visualization stage) in which similar miRNA–mRNA modules could be amalgamated. This would perhaps necessitate additional threshold settings. Also, it is possible that individual solutions may be further refined by performing an additional ‘pruning’ step. This can be carried out by the user at the visualization stage via two adjustable sliders. We have also demonstrated that unifying visualization and analysis within a single software application allows the user to rapidly explore and query large complex, otherwise overwhelming, data sets.

Currently this approach models static networks; however, such visualization may be extended to include an extra dimension, e.g. by the addition of animation, to support the modelling of dynamic networks and how the miRNA–mRNA modules are perturbed with the onset of disease. In the current context, we are only scratching the surface, and we envisage the development of a rich vein of research in the area of miRNA functional modules and their contribution to normal gene regulation and dysregulation in disease.

## SUPPLEMENTARY DATA

Supplementary Data are available at NAR Online.

## FUNDING

Funding for open access charge: Children’s Medical and Research Foundation, Dublin, Ireland, Science Foundation Ireland [07/IN.1/B1776] and the NIH [5R01CA127496].

*Conflict of interest statement*. None declared.

## Supplementary Material

Supplementary Data
