# Supplementary material for: Discovery and visualization of miRNA–mRNA functional modules within integrated data using bicluster analysis
Source: Nucleic Acids Res. 2013 Dec 18;42(3):e17. doi: 10.1093/nar/gkt1318 (PMC3919560; doi:10.1093/nar/gkt1318)
Supplement: Supplementary Data [file supp_42_3_e17__index.html]

Discovery and visualization of miRNA–mRNA functional modules within integrated data using bicluster analysis — Supplementary Data 

# Discovery and visualization of miRNA–mRNA functional modules within integrated data using bicluster analysis

## Supplementary Data

files

**Files in this Data Supplement:**

- Supplementary Data - xlsx file
- Supplementary Data - docx file
