# Supplementary material for: Discovery and visualization of miRNA–mRNA functional modules within integrated data using bicluster analysis
Source: Nucleic Acids Res. 2013 Dec 18;42(3):e17. doi: 10.1093/nar/gkt1318 (PMC3919560; doi:10.1093/nar/gkt1318)
Supplement: Supplementary Data [file supp_gkt1318_nar-01871-met-n-2012-File028.docx]

*Algorithm I: Seed Search.*

*Variable definitions: x : current solution,t_0_ : initial temperature, t : current temperature, rate : temperature fall rate, a : attempts, s : successes, a_count_ : attempt _count_, s_count_ : success _count_,,*

*F1 : fitness function(see footnote) , M : data matrix ,row : seed row size (mRNA), col : seed column size (miRNA)*

**Seed Search (t_0_, rate, row, col, s, a, M)**

**1. x = randomSolution(row,col)**

**2. t = t0**

**3. while(x not converging)**

**4. while(a_count_ < a AND s_count_ < s)**

**5. x_new_ = GenerateNewSolution(M, x)**

**6. if F_1_(x_new_) < F_1_(x)**

**7. then x = x_new_**

**8. else if exp(-ΔE/T )> random(0,1) (see footnote)**

**9. then x = x_new_**

**10. t = Cool(t,rate)**

**11. mask x from future seed searches (e.g. replace values with 0’s)**

**12. return x**

miRNA-mRNA module Fitness Function:

Probability of accepting a worse solution is proportional to the size dis-improvement and the current system temperature (i.e. Boltzman equation)

*Algorithm II: The Seed Expansion Phase.*

*Variable definitions:*

*r : row, c : column, R : rows in seed (mRNA), C : columns in seed (miRNA),*

*r_T_ : seed row threshold, c_T_ : seed column threshold.*

**SeedExpansion(Seed(R,C), rT,cT)**

**1. Get mean correlation of all r not in R**

**2. Get mean correlation of all c not in C**

**3. Sort r means**

**4. Sort c means**

**5. select r/c with best mean correlation (best = lowest (inverse) /or highest (positive))**

**7. if (r/c number < r/c_T_ ) AND (r/c mean correlation <= Seed Score)**

**8. add r/c to Seed(R,C)**

**9. re-score Seed(R,C)**

**10. return expanded Seed(R,C)**

**Building the Association Matrix – Selection of the *θ* correlation threshold**

*This is actually an interesting issue that we did not detail with fully in the paper. We looked at applying some correlation* θ *threshold, thinking that if we filtered for good correlations that we would improve the quality of our results. When we attempted to do so we found that (a) our results in terms of p-values disimproved (b) we saw less of a variety of functional modules.*

*We that this somewhat counter intuitive result may be explained by the fact that the data is inherently noisy and at often correlations that do not make the cut are needed to tie together larger better correlated parts of a module. So that a module would appear in this set split into two or more sub-modules when the* θ *was added. Additionally we think that this filtering based on a select correlation (*θ*) threshold also causes steep cliffs in the search space making it difficult for the optimization algorithm (even one employing Simulated Annealing that attempts to avoid local minima) to go from one good solution to another. This had the effect of only finding a limited variety of functional groups as some solution, although good, were just out of reach of the search.*
